# The Dunning–Kruger effect: subjective health perceptions on smoking behavior among older Chinese adults

**DOI:** 10.1186/s12889-023-16582-y

**Published:** 2023-09-04

**Authors:** Zhike Jia, Shubin Li, Zhihua Luo, Minjun Tong, Tianyue Gao

**Affiliations:** 1 School of Philosophy and Sociology, Hebei University, Baoding Hebei, China; 2 Asian Demographic Research Institute, Shanghai University, Shanghai, China; 3 School of Social Development, East China Normal University, Shanghai, China; 4 School of Foreign Languages and Business, Minjiang Teachers College, Fuzhou, China; 5 School of Economics, Hebei University, Baoding, Hebei China

**Keywords:** Dunning–Kruger effect, Subjective sense of health, Aging, Smoking behavior, Mediating effect

## Abstract

**Background:**

The intrinsic damage and external hazards of smoking are major risk factors for poorer health and are recognized as a global health issue of concern in geriatric health. This study aims to assess the Dunning–Kruger effect through the influence of subjective health perceptions on smoking behavior in older adults.

**Methods:**

This study used data from the 2018 Chinese Longitudinal Healthy Longevity Survey (*N* = 9,683) provided by the Center for Healthy Aging and Development Studies at Peking University. A binary logistic model was used to examine whether the Dunning–Kruger effect affects smoking behavior in older adults, and a linear probability model was used as a commentary baseline model for logistic regression to prevent measurement bias. In addition, a mediating analysis was used to examine the mechanisms through which the Dunning–Kruger effect occurs.

**Results:**

Older adults often overestimated their current health status and underestimated the health risks of smoking, causing the Dunning–Kruger effect to arise from their inadequate self-perceived health (i.e., older adults are more likely to smoke when they have better self-rated health or when hypertension, cardiopathy, stroke, and diabetes have little or no impact on their daily lives). These observations can be explained by the older adults’ subjective health perceptions arising from their ingenuous understanding of their health, which indirectly influences their smoking behavior to some extent.

**Conclusion:**

Older adults’ self-perceived health was associated with smoking behavior. Public health institutions should improve older adults’ health perceptions so that they objectively understand their own health status.

## Problem statement and research background

As the global population progressively ages, variations in the health status of the aging population and the causative mechanisms underlying such variations have garnered widespread attention from society and academia. Alongside its external hazards, the internal damage caused by smoking poses a significant health risk [1] and has emerged as an important global issue in geriatric health. A large body of empirical research has demonstrated that active smoking is detrimental to individual health since it increases the risk of cardiovascular disease [2, 3], chronic periodontal disease, [4] autoimmune disease, [5] coronary heart disease, [6] lung disease, and cancer [7]. Smoking is also closely associated with psychological behavior-related health effects such as anxiety, depression [8, 9], and attention-deficit/hyperactivity disorder [10]. Even if individuals choose not to actively smoke, they are still exposed to second-hand cigarette smoke, which is a risk factor for lung cancer and mortality, [11] cognitive impairments, and aggressive behavior [12]. These effects generally exacerbate the health burden of a nation’s population. Therefore, active and passive smoking damages an individual’s physical and mental health, with such damages often persistent, complex, and extensive.

While the aforementioned studies have effectively explained the impacts of smoking on individual health, they have mostly adopted a public health viewpoint to examine the health impacts of smoking in adolescents or women [13, 14]. Therefore, there is a lack of sociological investigation on the intrinsic association between self-perceived health and smoking behavior among older adults. In addition, the existing body of research has focused primarily on the health impacts of active or passive smoking [15] and overlooked the opposite effects of health status on smoking behavior. In particular, the mechanisms of action in which self-rated health restricts smoking behavior remain unknown. Therefore, analyzing these mechanisms is distinctive from the vast body of existing studies on the health impacts of smoking behavior and paves the way for reducing tobacco addiction through a holistic approach, thereby safeguarding public health.

In psychology, the Dunning–Kruger effect is a phenomenon of meta-ignorance, or ignorance of ignorance, which arises from a lack of professional knowledge, the concealment of knowledge in the realm of “unknown unknowns,” or ignorance being disguised by incorrect beliefs and background knowledge that seemingly appear as a correct answer [16, 17]. The Dunning–Kruger effect suggests that individuals may experience a cognitive bias in which they overestimate their own knowledge base. Compared to those with higher health literacy, those with lower health literacy had the same or greater confidence in health literacy, but experienced more problems engaging in health behaviors [18]. The mechanisms driving the Dunning-Kruger effect consist of two main types: One is the better than average effect, in which individuals overestimate their self-ratings when comparing themselves to their peers [19]; and the other is the false consensus effect, in which people overestimate how similar others are to themselves [20]. Currently, the Dunning-Kruger effect has been widely used in research in the areas of healthy choices [21], information literacy skills [22], intercultural competence (awareness) [23], intellectual ability [24], subjective financial literacy [25], and high-level reasoning [26].

In this study, we used the Dunning–Kruger effect to study older adults’ smoking behavior for two reasons. First, given that self-rated health is a subjective perception, older adults often distort or overestimate the expertise they lack but claim to have. Second, due to the inconspicuous nature of the health risks of tobacco, the Dunning–Kruger effect occurs in older adults regarding their self-perceived health. Therefore, this study seeks to address the following questions: Does the Dunning–Kruger effect influence the smoking behavior of older Chinese adults? How does the effect manifest itself in older smokers? If the effect does exist in older adults’ self-perceived health, through what mechanisms does it affect their smoking behavior? The influence of health on smoking behavior is multidimensional at an individual level. This influence may exist in two forms: (1) Smoking limits an older adult’s capacity to perform activities of daily living and instrumental activities of daily living, with these constraints including impaired mobility and poor health; [27] (2) The effects of self-rated health are more profound since some undiagnosed older patients may not display obvious symptoms during the early stage, and their ignorance of their illness may cause them to overestimate their actual health status [22]. Consequently, the Dunning–Kruger effect manifests as more frequent and intense smoking behavior. To better understand and monitor the influence of health status on older adults’ smoking behavior, this study focused on examining and estimating the influence of older adults’ self-perceived health on smoking behavior based on data from the 2018 Chinese Longitudinal Healthy Longevity Survey (CLHLS), and after controlling for individual and family backgrounds. The preliminary results present the mechanisms by which self-perceived health constrains smoking behavior in older adults, broadening our knowledge about future trends and patterns in the variation of older adults’ smoking behavior.

## Literature review and hypotheses

Existing research has used numerous theories to explain smoking behavior, including rational addiction, planned behavior, social identity, and cognitive dissonance theories. Rational addiction theory suggests that smoking behavior results from rational decision-making by individuals, a choice made after weighing their current benefits, future benefits, and the costs of smoking [28, 29]. The theory of planned behavior, which posits that perceived social norms influence behavior by affecting behavioral intentions [30], is also supported by a large body of empirical research [31]. Social identity theory suggests that an individual’s perception of the group to which they belong influences their self-concept, which in turn leads them to behave in a manner consistent with the norms of that group [32]. Regarding smoking behavior, different individuals who smoke are in different groups, and group norms or attitudes about smoking behavior influence an individual’s “smoker identity.” Studies have shown that individuals are more likely to identify as smokers when their peers approve of smoking [33]. Cognitive-dissonance theory explains smoking behavior from the perspective of identity, in that individual who perceive themselves as belonging to a certain identity may develop a state of cognitive dissonance if they do not engage in behaviors consistent with that identity. Therefore, most people usually engage in behaviors consistent with their identity [34]. One study found that the more people defined themselves as “smokers,” the more they smoked [35].

Existing theories generally explain the causal mechanisms of smoking behavior from different disciplinary perspectives. Rational addiction theory explains the occurrence of smoking decision-making behavior from the rational economic man assumption in economics. The theory of planned behavior explores the transmission process of smoking behavior among subjective norms, intentions, and behaviors from a psychological perspective. The social identity and cognitive-dissonance theories reveal the inherent relationship between individual smoking behavior and group attitudinal norms. While these theories provide a good reference for a better understanding and appreciation of the occurrence of smoking behavior, they remain inadequate in two aspects. Firstly, an individual’s decision to smoke is influenced by various factors, especially their own health. While some existing theories can be used to explain the influence of physical health on smoking behavior, they are limited by the heterogeneity of individual knowledge and experience and different individuals’ perceptions of their own health status. Secondly, most existing theoretical explanations of smoking behavior are based on a single disciplinary perspective. While this approach provides an opportunity to analyze and understand the causal mechanisms of smoking behavior in depth, it is challenging to observe the intrinsic connections between different theories and to test the magnitude of the effects of various factors on smoking behavior under different theoretical frameworks. Therefore, we have developed a new theoretical framework from a dual-disciplinary theoretical perspective of economics and sociology using the static exchange and dynamic addictive behavior theories to test the Dunning–Kruger effect” between subjective health and smoking behavior to enrich further the research findings in the smoking behavior field.

The exchange theory suggests that an individual’s decision whether or not to smoke or cease smoking is based on their comparison of the costs and benefits of smoking [36]. The benefits of smoking arise from the smoker’s subjective expectations, such as smoking reducing anxiety, bringing them closer to others, and making them more recognizable. Regarding costs, smokers must shoulder the economic, health-related, and social costs of passive smoking in others [37, 38]. According to exchange theory, the more benefits an individual gains from smoking, the more likely they are to smoke; the higher the cost of smoking, the less likely they would be to smoke. Therefore, in the decision-making between smoking and not smoking, when they perceive that the benefits of smoking outweigh its costs, they are more likely to smoke.

While the high returns and low cost of smoking may incite smoking behavior, older adults must consider additional factors in their decision to smoke, including their family’s health and economic burden and their children’s demands. Nevertheless, for older smokers, these economic and social costs are hardly determinants of their decision to smoke, which elevates the importance of the health-related costs of smoking. Firstly, while smoking harms an individual’s health, such health impacts are accompanied by a hysteresis effect in which the impacts of prolonged smoking since a smoker’s youth are only fully manifested in their older years [39]. This explains why older adults’ health status constrains their decision to smoke. Secondly, changes occur in an individual’s beliefs on the reasonability of smoking [40]. Older adults often deny the risks of smoking during their youth or perceive they can be offset through sports and healthy eating [41]. In other words, they subjectively perceive that the benefits of smoking outweigh its costs. However, as they age, they begin to deepen their knowledge of the harmful effects of smoking, changing their previous beliefs on the reasonability of smoking. Therefore, a psychological moderating effect occurs when they realize that the costs of smoking, especially the health-related costs, are significantly higher than they had initially believed. Lastly, changes in an individual’s health-related beliefs or views are also the main reason why older adults perceive the true costs and returns of smoking. Older adults often pay more attention to their health than their younger counterparts. The various health risks of smoking result in direct economic loss and functional impairment. Therefore, despite the wide range of returns that smoking confers for older adults, the health-related and other costs profoundly impact their smoking behavior. We propose the following two hypotheses:

- Hypothesis 1a: A Dunning–Kruger effect on an individual’s self-health awareness is more likely to occur in older adults with better self-rated health, resulting in the overestimation of their own health, the underestimation of the hazards of tobacco, and an increase in their likelihood of adopting smoking behavior.
- Hypothesis 1b: The Dunning–Kruger effect is more likely to occur in older adults whose activities of daily living are less affected by hypertension, cardiopathy, stroke, and diabetes (henceforth collectively referred to as the four diseases), resulting in the overestimation of their own health, the underestimation of the hazards of tobacco, and an increase in their likelihood of adopting smoking behavior.

Exchange theory explains how the costs and benefits of smoking relate to the decision to smoke. However, from a dynamic perspective, the theory of rational addiction further reveals the influence of an individual’s preference for smoking behavior. This theory suggests that most consumption behaviors are optimized when an individual has a stable preference [42]. Addiction does not alter an individual’s rationality, and many smokers are not necessarily short-sighted when they make their consumption decisions; instead, they can forecast the changes in cigarette prices and their impacts on cigarette buying [43–45]. However, when smokers are deeply addicted to cigarettes or their previous and current cigarette consumption behaviors are strongly complementary, their consumption becomes unstable. In addition, smoking behavior involves an individual’s interactions with cigarettes, which manifests as the short-term enjoyment and long-term benefits that smokers gain from smoking and the variation in smoking intensity caused by major life events. While a higher income and psychological stress stimulate an individual’s future desire for cigarettes, these temporary events also reduce the total utility while increasing the marginal utility of cigarette consumption [46]. For example, the psychological enjoyment of smoking is insufficient to offset a heightened risk of chronic diseases, higher mortality risk coefficients, and other health risks. Therefore, the influence of these events on cigarette consumption is extremely diminished. The theory of rational addiction offers a dynamic explanation for the changes in smoking consumption and embodies the mechanisms explaining changes in smoking intensity.

For older smokers, the longer their smoking duration and the older they are, the stronger their smoking habits. The intensity of their cigarette consumption is also often a steady state. Despite the detrimental effects of smoking on geriatric health, these effects are only extrinsic when combined with other behaviors such as tea consumption, alcohol consumption, and sleep quality. Research has shown that consuming tea improves health [47]. A causal relationship exists between alcohol and > 60 diseases, and 4% of the global disease burden can be attributed to alcohol consumption [48]. Sleep impairment is strongly associated with numerous long-term adverse physical and mental health outcomes, such as all-cause mortality, diabetes, and cardiovascular disease [49]. Therefore, the deterioration of health status and changes in their concept of health may alter an older smoker’s self-perceived health. The naïve or erroneous health perceptions established through an individual’s past daily life experiences may very well cause the Dunning–Kruger effect. Therefore, we propose the following two hypotheses based on the mechanisms through which self-perceived health causes the Dunning–Kruger effect.

- Hypothesis 2a: Self-rated health has an indirect or mediating effect on tea and alcohol consumption, sleep quality, and smoking behavior among older adults.
- Hypothesis 2b: Self-rated health has an indirect or mediating effect on hearing loss, visual acuity, and smoking behavior among older adults.
- Hypothesis 2c: Self-rated health has an indirect or mediating effect on the number of major diseases diagnosed and smoking behavior among older adults.

## Data, variables, and methods

### Data source

This study used data from the 2018 CLHLS provided by the Center for Healthy Aging and Development Studies at Peking University. The baseline and follow-up investigations in the CLHLS covered 23 provinces, autonomous regions, and municipalities in China, and the sample had good representativeness. Since this study focused on older adults aged ≥ 65 years, we removed individuals aged < 65 years from the sample during processing and analysis, followed by those with missing or no responses for the core variables. The final sample size in this study was 9683.

### Variable selection

The variable types used in this study were response, core explanatory, mediating, and control variables. Descriptive statistics of the variables are provided in Table 1.

**Table 1:** Descriptive statistics of the variables

Variable category	Variable and value	Mean	Standard deviation	Minimum	Maximum
Response variable	Smoking status (1 = smoker, 0 = non-smoker)	0.15	0.36	0	1
Explanatory variables	Self-rated health (1 = excellent, 2 = good, 3 = fair, 4 = poor, 5 = very poor)	2.54	0.89	1	5
	Degree to which the four diseases impact daily life (1 = severe, 2 = moderate, 3 = none)	2.51	0.63	1	3
Mediating variables	Daily intake (in units of 0.05 kg)	5.31	4.63	0	30
	Frequency of tea consumption (1 = daily, 2 = at least once weekly, 3 = at least once monthly, 4 occasionally, 5 = almost none or never)	4.07	1.60	1	5
	History of alcohol consumption	0.14	23.87	0	90
	Sleep quality (1 = excellent, 2 = good, 3 = fair, 4 = poor, 5 = very poor)	2.49	0.98	1	5
	Development of hearing difficulties (1 = very fast (several days), 2 = fast (several months), 3 = slow (several years), 4 = none or not hard of hearing)	3.61	0.58	1	4
	Visual acuity (1 = clear, 2 = basically clear, 3 = blurred, 4 = impaired)	1.48	0.77	1	4
	Number of major diseases diagnosed	0.45	1.62	0	80
Control variables	Age	83.79	11.46	65	117
	Sex (1 = male, 0 = female)	0.45	0.49	0	1
	Area of residence (1 = urban, 0 = rural)	0.58	0.49	0	1
	Household socioeconomic status, (1 = affluent, 2 = wealthy, 3 = average, 4 = poor, 5 = impoverished)	2.88	0.63	1	5
	Length of education received	3.59	4.34	0	29

The response variable was smoking behavior (*smoke*_*i*_), a binary variable obtained by querying whether the older respondents were current smokers.

The core explanatory variables were self-rated health (*health*_*i*_*)* and the degree to which the four diseases impacted the daily lives of older adults* (DADL*_*i*_*).* Self-rated health reflects the older respondents’ subjective perceptions of their own health and is an important indicator when measuring the Dunning–Kruger effect. In the CLHLS, self-rated health comprised five levels: extremely good, good, fair, poor, and extremely poor. The degree to which the four diseases impacted the daily lives of older adults further describes the Dunning–Kruger effect on the health perceptions of older adults. The reasons for adopting this variable were as follows. First, these four diseases are very common among older adults. Second, they have high flexibility in treatment, meaning they can be controlled through medication compliance, diet adjustment, and adequate training. Lastly, specialists generally evaluate and diagnose these four diseases, thus offering better objectivity. The variation in these four diseases can better reflect the older adults’ degree of perception toward and the importance they attached to disease and their subjective health status. In this study, the degree to which these four diseases impacted the older adults’ daily lives included severe, moderate, and no influence.

Mediating variables (*X*_*i*_): Most older adults do not have professional medical knowledge because they are not doctors. In addition, their understanding of their own health is often based on perceptions or impressions, which could be due to their habit of not attending health examinations regularly. Therefore, this study selected six mediating variables to measure the health status of older adults: self-reported hearing (not hard of hearing) and visual acuity (no dim vision), food intake (able to eat), frequency of tea consumption, history of alcohol consumption (able to drink), sleep quality (able to sleep), and the number of major diseases diagnosed (not diagnosed).

Control variables (*C*_*i*_): Based on previous studies, this study selected age, sex, length of education received, household socioeconomic status, and area of residence (urban/rural) as control variables. Existing studies have shown that age, sex, urban–rural differences, household socioeconomic status, and length of education received significantly affect smoking behavior. This study selected the aforementioned variables as control variables to better examine the influence of self-perceived health on smoking behavior in older adults and to accurately reflect the net effects of self-perceived health. The preconditions for smoking include cigarette buying and household socioeconomic status (affluent, wealthy, average, poor, or impoverished), which may influence an older adult’s tobacco buying and the type of tobacco purchased. Therefore, household socioeconomic status was included as a control variable to reflect their economic behavior through smoking.

### Statistical methods and analytical strategies

First, to examine whether the Dunning–Kruger effect affects smoking behavior in older adults, a binary logistic model was used to analyze the features of the response variable.

1 $$In\left(\frac{{Smoke}_{i}}{{1-Smoke}_{i}}\right)={\beta }_{0}+{\beta }_{1}{SRH}_{i}+{\beta }_{2}{Z}_{i}+{\varepsilon }_{i}$$

In Eq. (1), *Smoke*_*i*_ represents the smoker status of the *i*^th^ older adult, *SRH*_*i*_ represents the self-rated health of the *i*^th^ older adult (excellent, good, fair, poor, or very poor), *Z*_*i*_ is a control variable (age, sex, length of education received, area of residence, or household socioeconomic status), *β*_0_ is a constant, *β*_1_ and *β*_2_ are parameters to be evaluated, and *ɛ*_i_ is the error term.

To further examine the influence of the Dunning–Kruger effect on smoking behavior in older adults from a chronic disease perspective, this study examined how smoking behavior was influenced by the degree to which the four diseases impacted the daily lives of older adults.

2 $$ln\left(\frac{{Smoke}_{i}}{1-{Smoke}_{i}}\right){\varphi }_{0}+{\varphi }_{1}{DADL}_{i}+{\varphi }_{2}{Z}_{i}+{\omega }_{i}$$

In Eq. (2), *Smoke*_*i*_ represents the smoker status of the *i*^th^ older adult, *DADL*_*i*_ represents the degree to which the four diseases impact the daily life of the *i*^th^ older adult (severe, moderate, or none), *Z*_*i*_ is a control variable (age, sex, length of education received, area of residence, or household socioeconomic status), $\varphi_0$ is a constant, $\varphi_1$ and $\varphi_2$ are parameters to be evaluated, and ${\omega }_{i}$ is the error term.

In order to overcome the problem of unobserved heterogeneity that exists because the regression coefficients in a logistic regression model cannot be directly compared, this study used a linear probability model (LPM) as a commentary baseline model for logistic regression to prevent measurement bias. Since endogeneity may exist between the independent variables (self-rated health and the degree to which the four diseases impact daily life) and the dependent variable (smoking status), this study also adopted an instrumental solution to address endogeneity.

This study used a mediating analysis to examine the mechanisms through which the Dunning–Kruger effect occurs. However, since the response variable was binary and a wider body of research is available on testing the mediating effects when it is categorical, this study had to perform specific judgments and selections due to the advantages and disadvantages of each method. Mackinnon and Cox suggested the product distribution method to analyze mediating effects of a categorical variable based on the significance of Z_a _× Z_b_ [50]. The advantage of this method is that it unifies linear and logistic regressions, generating more realistic mediating effects. However, its drawback is that existing statistical software cannot provide the bootstrap confidence interval in the product distribution, and, similar to structural equation modeling, some measurement errors also exist. When examining mediating effects through structural equation modeling, the Probit function is used as a link function to adjust the variance of the mean in the weighted least squares using the mean and variance approach. This approach generates the bootstrap confidence interval and eliminates measurement errors. However, since all of its pathways are linked using the Probit function, bias may exist in the mediating effect testing caused by regression methods. Therefore, we simultaneously used the product distribution approach and structural equation modeling to validate mediating effects, obtaining more robust test results. The criteria for evaluating the goodness of the fit of the structural equation model include a root mean square error of approximation < 0.05, a comparative fit index > 0.9, and a standardized root mean square residual < 0.08 [51].

## Analysis of the main results

### The Dunning–Kruger effect in the smoking behavior of older adults

In this study, all the observed variables were subjected to logistic regression analysis (see Table 2– Self-rated health). However, since the logistic regression coefficients cannot be compared, we used the LPM model for comparison instead (see Table 2– Self-rated health [5]). Excellent and good self-rated health were rearranged into a new set of observed values termed “self-rated as good” (see Table 2– Self-rated as good), fair self-rated health was still termed “self-rated as fair” (see Table 2– Self-rated as fair), and poor and very poor self-rated health were rearranged into a new set of observed values termed “self-rated as poor” (see Table 2– Self-rated as poor).

**Table 2:** The influence of self-rated health on smoking behavior in older adults

Variable	Full model	Sub-model		
	Self-rated health (1) (*Logit*)	Self-rated as good (2) (*Logit*)	Self-rated as fair (3) (*Logit*)	Self-rated as poor (4) (*Logit*)
Self-rated health	-0.112** (0.894)	0.253*** (1.288)	-0.205** (0.815)	-0.121 (0.415)
Control variable	YES			
Observed value	10,095	10,095	10,095	10,095
**Cox *R***^***2***^	0.129	0.129	0.129	0.128
Variable	Full model	Sub-model		
	Self-rated health (5) (*LPM*)	Self-rated as good (6) (*LPM*)	Self-rated as fair (7) (*LPM*)	Self-rated as poor (8) (*LPM*)
Self-rated health	-0.034***	0.040***	-0.027**	-0.018*
Control variable	YES			
Observed value	10,095	10,095	10,095	10,095
**R**^**2**^	0.129	0.129	0.128	0.128

Bracketed values in the logistic model are odds ratios; *, *p* < 0.05; **, *p* < 0.01; ***, *p* < 0.001

In Table 2, Self-rated health (1) and (5) represent the logistic and LPM models of all observed values, respectively. The two models’ predicted results were similar since the regression coefficients were all negative and significant at the 1% and 0.1% levels (Logit: *β* =  − 0.112, *p-value* < 0.01; LPM: *β* =  − 0.032, *p-value* < 0.001). The two models suggest that older adults are more likely to smoke when they have better self-rated health.

In the sub-models, Self-rated as good had the same coefficients and were significant at the 0.1% level (Logit: *β* = 0.253, *p-value* < 0.001; LPM: *β* = 0.040, *p-value* < 0.001). The two models suggest that older adults who rated their health as good are more likely to smoke than those who rated otherwise. Specifically, older adults with good self-rated health are 1.28 times more likely to smoke than those with poor self-rated health. A marked increase in the regression coefficients was observed from Self-rated health (5) to Self-rated as good (6). The regression coefficients for Self-rated as fair (3) and (7) were negative and significant at the 1% level (Logit: *β* =  − 0.205, *p*-*value* < 0.001; LPM: *β* =  − 0.027, *p*-*value* < 0.001), suggesting that fair self-rated health is a protective factor against smoking behavior. Older adults with fair self-rated health had a considerably lower likelihood of smoking than those with other self-rated health ratings. The regression coefficients for Self-rated as poor (4) and (8) were negative. While the Logit regression coefficient was nonsignificant (*β* =  − 0.121), the LPM model was significant but with a lower significance level (*β* =  − 0.018, *p-value* < 0.05), suggesting that poor self-rated health may be a protective factor against smoking behavior.

Based on the consolidated results of the logit and LPM models of self-rated health presented in Table 2, older adults are more likely to smoke when they have better self-rated health. This finding suggests that some older adults overlook the health risks of tobacco when they subjectively perceive that there are no remarkable differences in their current health. Therefore, we demonstrated that the Dunning–Kruger effect occurs in smoking behavior in older adults, validating Hypothesis 1a.

Next, we examined whether the Dunning–Kruger effect exists from the perspective of the impacts of chronic diseases on smoking behavior in older adults. All the observed values were subjected to logistic regression analysis (see Table 3– Degree of impact) and compared using the LPM model (see Table 3– Degree of impact). A severe impact of the four diseases on daily life was rearranged into a new set of observed values termed “Severe”, a moderate impact was termed “Moderate”, and no impact was termed “None”.

**Table 3:** The influence of the impact of diseases on smoking behavior in older adults

Variable	Full model	Sub-model		
	Degree of impact (1) (*Logit*)	Severe (2) (*Logit*)	Moderate (3) (*Logit*)	None (4) (*Logit*)
Degree of impact	0.290*** (1.336)	-0.617*** (0.539)	-0.169** (0.844)	0.274*** (1.316)
Control variable	YES			
Observed value	9683	9683	9683	9683
**Cox *R***^***2***^	0.129	0.130	0.129	0.128
Variable	Full model	Sub-model		
	Degree of impact (5) (*LPM*)	Severe (6) (*LPM*)	Moderate (7) (*LPM*)	None (8) (*LPM*)
Degree of impact	0.057***	-0.044***	-0.026**	0.044***
Control variable	YES			
Observed value	9683	9683	9683	9683
***R***^***2***^	0.130	0.130	0.129	0.130

Bracketed values in the logistic model are odds ratios; *, *p* < 0.05; **, *p* < 0.01; ***, *p* < 0.001

Table 3 shows that the coefficients for Degree of impact (1) and (5) were positive and significant at the 0.1% level (Logit: *β* = 0.290, *p*-*value* < 0.001; LPM: *β* = 0.057, *p*-*value* < 0.001), suggesting that older adults are more likely to smoke when the four diseases had a lower or no impact on their daily lives. The coefficients for Severe (2) and (6) were negative and significant at the 0.1% level (Logit: *β* =  − 0.617, *p*-*value* < 0.001; LPM: *β* =  − 0.044, *p*-*value* < 0.001), suggesting that older adults are less likely to smoke when the four diseases had a larger impact on their daily life. The severity of the impacts implies that smokers should cease smoking. Similar conclusions were drawn from the Moderate (3) and (7) models. Interestingly, the coefficients of None (4) and (8) were positive and significant at the 0.1% level (Logit: *β* = 0.274, *p*-*value* < 0.001; LPM: *β* = 0.044, *p*-*value* < 0.001), suggesting that older adults are more likely to smoke when the four diseases have no impact on their daily life than when they have some impact. This finding also indirectly implies that even when diagnosed with a disease, its impact on older adults’ smoking behavior was low when they subjectively perceived minimal or no impact.

According to the consolidated results of the logit and LPM models presented in Table 3, older adults are more likely to smoke when the four diseases have a lower impact on their daily life. Even when some of the older adults were diagnosed with these diseases, they would still overlook the risks of their illness when the subjectively perceived impacts were below a certain level. Therefore, the Dunning–Kruger effect occurs in those with good subjective self-perceived health who continued their smoking behavior, validating Hypothesis 1b.

### Addressing and handling endogeneity

A complementary causal relationship may exist between current smoking behavior and self-rated health. This study adopted “whether natural water was consumed as drinking water during childhood” as an instrumental variable for the following reasons. Firstly, most older adults in China have experienced the process of transitioning from drinking natural to tap water, and some studies have reported that tap water usage among older adults in China reached 81.6%, and tap water has health benefits for older adults [52]. Some studies have also reported that some risk of pharmaceutical residues exists in tap water production in China [53]. This would allow older adults to compare the difference between the natural water they had in childhood and the tap water they currently use. While this comparison is a subjective difference in perception, it could impact older adults’ self-rated health. Secondly, whether or not tap water was retrofitted depends mainly on the local government’s promotion of public infrastructure renewal and is unrelated to the older adult-centered variables used in this study [54].

First, most older adults in China drank natural water (spring or well water) in childhood. Next, drinking water in China is mainly converted from natural water to tap water, and older adults develop subjective perceptions about the quality of drinking water, such as a dislike of tap water due to chemical filtering and processing. Lastly, the value of and preference for natural or tap water may affect self-perceived health among older adults due to the mineral-rich composition and sweeter taste of natural water. However, regardless of whether natural or tap water is consumed, high drinking water quality standards are still attained when the water is boiled correctly, and there is no direct relationship between water quality and diseases, genes, and other omitted or psychological variables.

Since this study used LPM and Logit models to examine the relationship between self-perceived health and smoking behavior in older adults, the two-stage least-squares (2SLS) and IVprobit approaches were used to validate the endogeneity (see Table 4). The robust chi-squared value was 4.776 (*p-value* = 0.028), and the robust regression *F*-value was significant at the 5% level (*p-value* = 0.028), suggesting strong endogeneity. Table 4 shows that after 2SLS regression using the instrumental variable, self-rated health remained significant (*β* =  − 0.162*), suggesting that self-rated health significantly influenced smoking behavior even after addressing endogeneity. Additionally, the *F*-value was 26.91 after a weak correlation analysis of the instrumental variable, indicating the absence of a weak instrument.

**Table 4:** Analysis of endogeneity in the relationship between self-rated health and smoking behavior in older adults

Independent variable (2SLS)	B	Standard error	Significance
Self-rated health	-0.162	0.073	0.027
Control variable	YES		
Independent variable (IVprobit)	B	Standard error	Significance
Self-rated health	-0.697	0.224	0.002
Control variable	YES		

In an objective examination of subjective self-perceived health, we introduced the instrumental variable “availability of retirement pension” to mitigate endogeneity. The provision of a retirement pension scheme is primarily subject to the macroeconomic regulation and control policies of local and central governments and is less correlated with personal willingness (see Table 5). The robust chi-squared value (10.413) and the robust regression *F*-value were both significant at the 1% level (*p-value* = 0.001), suggesting strong endogeneity. After using the instrumental variable, the variation in the degree of impact of the diseases on daily life was significant at the 0.1% level, suggesting that the degree of impact of the diseases on daily life significantly constrained smoking behavior, even after addressing endogeneity. An *F*-value of 96.67 was obtained when examining the strength of the instrumental variable, indicating the absence of a weak instrument.

**Table 5:** The relationship between the degree of impact of diseases and smoking behavior in older adults

Independent variable (2SLS)	B	Standard error	Significance
Degree of impact	0.211	0.058	0.000
Control variable	YES		
Independent variable (IVprobit)	B	Standard error	Significance
Degree of impact	0.211	0.055	0.000
Control variable	YES		

### Validating the mechanisms through which the Dunning–Kruger effect mediates smoking behavior in older adults

After confirming that the Dunning–Kruger effect occurs in the smoking behavior of older adults, the relevant issues that must be addressed include the factors influencing their self-perceived health (and thus their smoking behavior), the means through which older adults subjectively perceive their health status, and whether these methods of subjective perception influence their smoking behavior. This study used a mediating analysis to validate the mediating mechanisms of the Dunning–Kruger effect regarding self-perceived health.

Since the product distribution and bootstrapping methods have their strengths and drawbacks, this study used both product distribution and structural equation modeling to leverage the advantages of both methods [55]. When testing mediating effects using the product distribution method, a regression analysis is first performed on the influence of the independent variables on the dependent variable to draw conclusions based on the significance of the direct effects. If the direct effects are not significant, then the conclusions should be drawn from the indirect effects. The mediating effects are deemed to exist if the direct effects are significant and the test results obtained through the R statistical software’s *RMediation* package are acceptable (a value of 0 is not included in a 95% confidence interval). When examining the mediating effects through structural equation modeling, besides the same assumptions established in the product distribution method, a value of 0 is excluded from a 95% confidence interval in bootstrapping.

As shown in Table 6, logistic regression of the independent variable on the dependent variable (whether or not to smoke) identified three variables that were not significant—staple food intake (*β* = 0.006), rate of development of hearing difficulties (*β* =  − 0.030), and visual acuity (*β* =  − 0.009)—indicating that they did not directly affect the decision whether or not to smoke. However, four variables significantly affected the decision whether or not to smoke: frequency of tea consumption (*β* =  − 0.139* **), history of alcohol consumption (*β* = 0.017, *p-value* < 0.001), sleep quality (*β* =  − 0.063, *p-value* < 0.05), and number of major diseases diagnosed (*β* =  − 0.090**). In addition, regression analysis of the independent variables on the mediating variable (self-rated health) revealed that only a history of alcohol consumption the number of years of alcohol consumption did not significantly affect self-rated health; the remaining six variables all significantly affected self-rated health. This finding suggests that a history of alcohol consumption may directly affect the choice of smoking behavior without an indirect effect.

**Table 6:** Regression analyses of the mediating effects

Independent variable → Dependent variable (Smoking status)	B	Standard error	Independent variable → Mediating variable (Self-rated health)	B	Standard error
Food intake	0.006	0.004	Food intake	-0.006***	0.002
Frequency of tea consumption	-0.139***	0.017	Frequency of tea consumption	0.019**	0.006
History of alcohol consumption	0.017***	0.001	History of alcohol consumption	0.000	0.000
Sleep quality	-0.063*	0.031	Sleep quality	0.275***	0.009
Development of hearing difficulties	-0.030	0.055	Development of hearing difficulties	-0.086***	0.016
Visual acuity	-0.009	0.044	Visual acuity	0.123***	0.012
Number of major diseases diagnosed	-0.090**	0.032	Number of major diseases diagnosed	0.056***	0.005
Control variable	YES				

^*^, *p* < 0.05; **, *p* < 0.01; ***, *p* < 0.001

Table 7 shows the results of the mediating analysis derived from the R statistical software’s *Mediation* package. The results indicate that a value of 0 was excluded from the 95% confidence intervals of all variables except a history of alcohol consumption, suggesting that indirect effects may exist. However, based on the conclusions presented in Table 6, food intake, the development of hearing difficulties, and visual acuity had no direct effects on smoking status. Therefore, we can only determine that these are indirect instead of mediating effects. Frequency of tea consumption, sleep quality, and the number of major diseases diagnosed passed both the direct and indirect effect tests and were thus determined to be mediating effects.

**Table 7:** Product distribution analysis of the mediating effects

Independent variable	95%LLCI	95%ULCI	Unstandardized coefficient	Standard error	*p*-value
Food intake	0.0002	0.0012	0.001	0.000	0.001
Frequency of tea consumption	-0.0036	-0.0006	-0.002	0.000	0.003
History of alcohol consumption	0.0000	0.0000	0.000	0.000	0.368
Sleep quality	-0.0436	-0.0109	-0.030	0.004	0.006
Development of hearing difficulties	0.0046	0.0163	0.009	0.001	0.001
Visual acuity	-0.0192	-0.0049	-0.013	0.001	0.001
Number of major diseases diagnosed	-0.0088	-0.0022	-0.006	0.002	0.005

Table 8 shows the results of the mediating effects analysis using structural equation modeling with 5000 bootstraps. While the results are similar to those obtained through product distribution, some differences exist. The results generated with the R statistical software’s *Mplus* package are expressed to three decimal places, while those generated with the R statistical software are expressed to four decimal places (see Table 7). The first three decimal points for food intake and frequency of tea consumption were 0, and a number only appeared after the fourth decimal point. However, as shown in Table 6, since only three decimal points were retained, a value of 0 was observed in the 95% confidence intervals, suggesting indirect effects, albeit marginal.

**Table 8:** Bootstrapping (5000 times) analysis of the mediating effects

Independent variable	95%LLCI	95%ULCI	Unstandardized coefficient	Standard error	*p*-value
Food intake	0.000	0.002	0.001	0.000	0.005
Frequency of tea consumption	-0.002	0.000	-0.001	0.000	0.028
History of alcohol consumption	0.000	0.002	0.000	0.000	0.434
Sleep quality	-0.027	-0.005	-0.016	0.006	0.004
Development of hearing difficulties	0.001	0.005	0.006	0.002	0.005
Visual acuity	-0.013	-0.004	-0.008	0.003	0.001
Number of major diseases diagnosed	-0.007	-0.001	-0.003	0.002	0.048

The higher the frequency of tea consumption, the better the sleep quality, and the fewer the number of major diseases diagnosed, the higher an individual’s self-rated health, further increasing the likelihood of smoking. Unlike indirect effects, these three mediating variables also directly affected the dependent variable. Regarding indirect effects, older adults with a higher food intake, slower or no hearing difficulty development, and better visual acuity had higher self-rated health. Unlike mediating effects, food intake, the development of hearing difficulties, and visual acuity indirectly affected smoking behavior in older adults through self-rated health but did not directly affect the dependent variable. This finding implies that older adults are more likely to smoke when they have better self-rated health, validating Hypotheses 2a, 2b, and 2c.

To summarize, based on the aforementioned empirical results, the older adults included in this study had an ingenuous understanding of self-rated health, which was partially based on their subjective perceptions. Admittedly, the general perception of an individual’s health can, to some extent, be reflected in their ability to eat, drink, and sleep; and the absence of hearing impairment, dim vision, and major diseases. These subjective judgments are crude and haphazard and lack accuracy and rationality. However, these ingenuous perceptions had improved older adults’ subjective judgments, resulting in the Dunning–Kruger effect in their self-perceived health, which caused them to overlook the health hazards and risks of smoking.

**Fig. 1:**
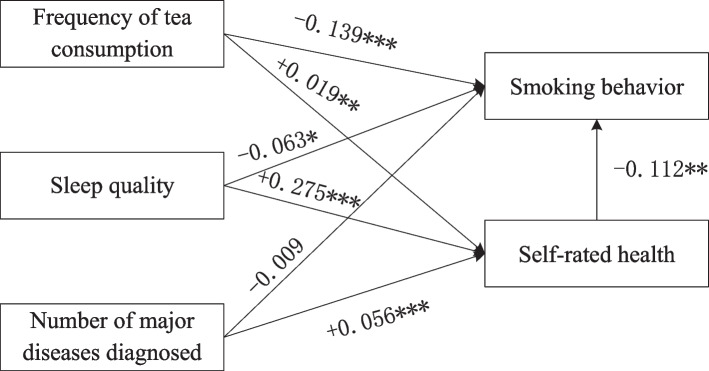
Path coefficients and significance levels

From the perspective of mediating effects, while the independent variables directly influenced the dependent variable without the assistance of the mediating variables, the indirect effects could only be achieved through the mediating variables, including the frequency of tea consumption, sleep quality, and the number of major diseases diagnosed (see Fig. 1). These variables are characterized by their large-scale but ambiguous or unspecific influence on older adults’ global perceptions of their health. For example, they could not explain the specific health benefits of tea drinking and instead provided conventional and multidimensional responses such as tumor suppression and hypertension prevention; this was also the case for sleep quality and the diagnosis of major diseases. Notably, poorer sleep quality affects older adults in many ways, including their social interactions, emotions, and cognitive abilities, while the diagnosis of major diseases impacts them physically and mentally. Given the ambiguous and extensive nature of these effects, older adults would intuitively generate a broad attribution toward adverse outcomes. Smoking is highly likely to become an attribution, thereby generating mediating effects. Older adults also generated a broad and conventional understanding of those behavioral factors with nonspecified effects (e.g., tea drinking). In other words, their vague understanding of the benefits of tea drinking resulted in a lax perception of the effectiveness of tea drinking in reducing the risks of smoking.

## Discussion

As expected, our study found that self-perceived health was associated with smoking behavior in older adults, consistent with the findings of existing studies [56]. Our results showed that older adults are more likely to smoke when they have better self-rated health or when hypertension, cardiopathy, stroke, and diabetes have little or no impact on their daily life. Some studies have reported that subjective health is a good predictor of all-cause mortality and disease prognosis in older adults and has a profound significance on public health policy formulation and health statistics surveying. In this context, subjective health can be reliable, generating the argument that an individual can be aware of their own illness and the changes to their health based on their own subjective health perceptions [57].

However, some studies have corrected the deficits of subjective health, arguing that subjective health hardly reflects the impacts of lifestyle habits on health, such as prolonged sitting and being overweight, suggesting that an individual can be unaware of their own illness [58, 59]. Indeed, subjective health offers many advantages as an indicator, such as elucidating the future trends in geriatric health in an effortless and less time-consuming manner. However, our study also highlights the drawbacks of subjective health since it does not reflect the health risks of smoking for older adults. The Dunning–Kruger effect provides a possible theoretical path for testing the validity of subjective health indicators to some extent.

The older adults’ self-perceived health arises from their ingenuous understanding of health concepts, consistent with the theory of planned behavior’s perceived subjective norm-intention-behavior explanatory path. In the public health field, smoking is often described as a separation of knowledge and action. Epidemiological studies have specifically shown that while people are often aware that smoking harms their health, they are often unable to comply with anti-smoking behaviors in real life, leading to willful blindness or the separation of knowledge and action. However, our study found that the “knowledge” of older adults was not based on theory (e.g., smoking is harmful to health) but rather on subjective health perceptions. In other words, the decision of an older adult to smoke is based on their current subjective health perceptions. From this perspective, their smoking behavior is a unity of knowledge and action.

Our results also showed that older adults often overestimate their current health status and underestimate the health risks of smoking, causing the Dunning–Kruger effect to arise from their inadequate self-perceived health, which causes them to misbelieve that smoking has lower health costs. This finding also implies that the cost–benefit logic of exchange theory is valid in the relationship between subjective health and smoking behavior in older adults. However, some current public policies are based on the conclusion that smoking harms health, with little attention paid to the opposite effect of self-health perceptions and awareness effects on smoking behavior. In this respect, our study fills this gap to some extent and has enlightening implications for developing public policies related to smoking behavior.

The key contribution of our study is that, from the perspective of interdisciplinary exchange and addiction theories, we explain the effect of subjective health perception overestimation on smoking behavior (i.e., the Dunning–Kruger effect of an individual’s self-perception of their health status on smoking behavior), which helps to provide a basis for the formulation of a theory of smoking behavior appropriate to smokers. However, it is important to emphasize that our study complements and deepens previous theoretical explanations rather than develops an entirely new theory. The findings of our study also have implications for the development of tobacco intervention or control efforts in individual countries. Public health institutions should improve the health perceptions of older adults so that they objectively understand their own health status. The health impacts of tobacco on geriatric health could be reduced by instilling correct knowledge of its health risks among older adults.

## Limitations and future research directions

The main strength of our study is its clarification of the Dunning–Kruger effect and the mechanisms through which it mediates smoking behavior in older Chinese adults. However, our study had several limitations that need to be addressed. First, we used cross-sectional data from 2018. The lack of validation based on long-term data easily neglects the impacts arising from individual differences. Future longitudinal studies are needed to validate our findings. Second, our study failed to robustly explain why a history of alcohol consumption did not mediate smoking status through self-rated health. Further theories should be explored to explain the relationship between smoking behavior and self-health. Third, our study performed a mediating analysis using a stepwise approach, and we hope to use better approaches for validating the mediating effects in future studies. Lastly, we only focused on the subjective health perceptions of older adults toward smoking. Therefore, more in-depth and precise research should be performed on the Dunning–Kruger effect in older adults’ subjective health perceptions to facilitate an in-depth discussion.

## Conclusions

Our study provides additional evidence that older adults’ self-perceived health is associated with smoking behavior. Older adults often overestimate their current health status and underestimate the health risks of smoking, causing the Dunning–Kruger effect to arise from their inadequate self-perceived health. Therefore, public health institutions should improve the health perceptions of older adults so that they objectively understand their own health status in the future.

## Data Availability

The data used in this study can be available upon request. Please contact Pro. Jia at hbujzk@vip.163.com
